# Supplementary material for: Transperineal Laser Ablation of the Prostate (TPLA) for Lower Urinary Tract Symptoms Due to Benign Prostatic Obstruction
Source: J Clin Med. 2023 Jan 19;12(3):793. doi: 10.3390/jcm12030793 (PMC9918261; doi:10.3390/jcm12030793)
Supplement: Supplementary file 1 [file jcm-12-00793-s001.zip › Table S1.pdf]

Table S1. Risk of bias assessment according to the Quality in Prognosis Studies (QUIPS) tool.

Number	Report	**Reporting and Quality in Prognosis Studies (QUIPS) tool -** ☺Low Risk; ☹High Risk; ○ Moderate Risk					
		**QUIPS tool: domain 1** (study participation)	**QUIPS tool: domain 2** (study attrition)	**QUIPS tool: domain 3** (prognostic factor measurement)	**QUIPS tool: domain 4** (outcome measurement)	**QUIPS tool: domain 5** (study confounding)	**QUIPS tool: domain 6** (statistical analysis and reporting)
1	Sessa et al.	☺	☺	n/a	☺	○	☹
2	Cai et al.	☺	☺	n/a	○	☹	○
3	De Rienzo et al.	☺	☺	n/a	☺	○	○
4	Frego et al.	☺	☹	n/a	○	☹	○
5	Manenti et al.	☺	☺	n/a	☺	☹	○
6	Pacella et al.	☺	☺	n/a	○	☹	○
7	Patelli et al.	○	☺	n/a	○	☹	○
