# Supplementary figures and images for: Transperineal Laser Ablation of the Prostate (TPLA) for Lower Urinary Tract Symptoms Due to Benign Prostatic Obstruction
Source: J Clin Med. 2023 Jan 19;12(3):793. doi: 10.3390/jcm12030793 (PMC9918261; doi:10.3390/jcm12030793)

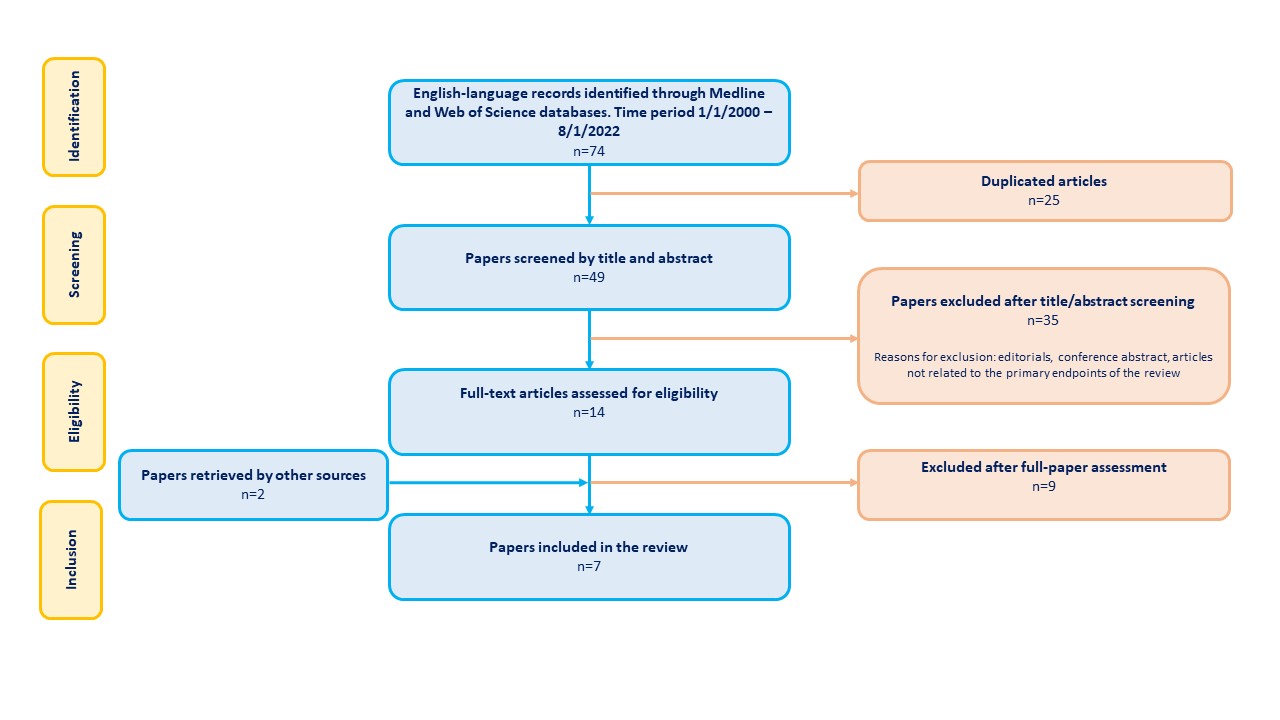

Supplement: Supplementary file 1 [file jcm-12-00793-s001.zip › Figure S1.jpg]
